# Deep learning for automatic organ and tumor segmentation in nanomedicine pharmacokinetics

**DOI:** 10.7150/thno.90246

**Published:** 2024-01-01

**Authors:** Alex Dhaliwal, Jun Ma, Mark Zheng, Qing Lyu, Maneesha A. Rajora, Shihao Ma, Laura Oliva, Anthony Ku, Michael Valic, Bo Wang, Gang Zheng

**Affiliations:** 1 Princess Margaret Cancer Centre, University Health Network, 101 College Street, Toronto, M5G 1L7, Ontario, Canada.; 2 Department of Medical Biophysics, University of Toronto, 101 College Street, Toronto, M5G 1L7, Ontario, Canada.; 3 Department of Laboratory Medicine and Pathobiology, University of Toronto, 1 King's College Circle, Toronto, M5S 1A8, Ontario, Canada; 4 Peter Munk Cardiac Centre, University Health Network, 190 Elizabeth St, Toronto, M5G 2C4, Ontario, Canada.; 5 Department of Computer Science, University of Toronto, 101 College Street, Toronto, M5G 1L7, Ontario, Canada.; 6 Institute of Biomedical Engineering, University of Toronto, 101 College Street, Toronto, M5G 1L7, Ontario, Canada.; 7 Vector Institute for Artificial Intelligence, 661 University Avenue, Toronto, M4G 1M1, Ontario, Canada.; 8 Techna Institute, University Health Network, 190 Elizabeth Street, Toronto, M5G 2C4, Ontario, Canada.; 9 Department of Radiology, Stanford University, 1201 Welch Road, Stanford, 94305-5484, California, United States of America.

**Keywords:** Deep Learning, Nanomedicine, Pharmacokinetics, Auto-Segmentation, Radioimaging, Functional Imaging, Multimodal, Multiparameter, Contouring, PET, CT

## Abstract

**Rationale**: Multimodal imaging provides important pharmacokinetic and dosimetry information during nanomedicine development and optimization. However, accurate quantitation is time-consuming, resource intensive, and requires anatomical expertise.

**Methods**: We present NanoMASK: a 3D U-Net adapted deep learning tool capable of rapid, automatic organ segmentation of multimodal imaging data that can output key clinical dosimetry metrics without manual intervention. This model was trained on 355 manually-contoured PET/CT data volumes of mice injected with a variety of nanomaterials and imaged over 48 hours.

**Results**: NanoMASK produced 3-dimensional contours of the heart, lungs, liver, spleen, kidneys, and tumor with high volumetric accuracy (pan-organ average %DSC of 92.5). Pharmacokinetic metrics including %ID/cc, %ID, and SUV_max_ achieved correlation coefficients exceeding R = 0.987 and relative mean errors below 0.2%. NanoMASK was applied to novel datasets of lipid nanoparticles and antibody-drug conjugates with a minimal drop in accuracy, illustrating its generalizability to different classes of nanomedicines. Furthermore, 20 additional auto-segmentation models were developed using training data subsets based on image modality, experimental imaging timepoint, and tumor status. These were used to explore the fundamental biases and dependencies of auto-segmentation models built on a 3D U-Net architecture, revealing significant differential impacts on organ segmentation accuracy.

**Conclusions**: NanoMASK is an easy-to-use, adaptable tool for improving accuracy and throughput in imaging-based pharmacokinetic studies of nanomedicine. It has been made publicly available to all readers for automatic segmentation and pharmacokinetic analysis across a diverse array of nanoparticles, expediting agent development.

## Introduction

Preclinical nanomedicine development relies upon accurate interpretation of pharmacokinetic data. Although longitudinal imaging studies can reduce the time and resource burden associated with developing novel agents, optimization across the multitude of parameters that influence agent circulation and biodistribution (formulation, dosage, time frame, experimental model, etc.) quickly cause studies to exponentially increase in size and cost. For experiments that extract quantitative data from imaging techniques such as PET, SPECT, or whole-body fluorescence, manually generating contours for specific organs of interest is often excluded outright due to the massive investment of time and requirement to operate within inflexible, proprietary imaging software. In aggregate, these obstacles contribute to the vast under-utilization of informative preclinical imaging data and force researchers to subsist on simplified — and often incorrect 1,2 — representations of their pharmacokinetic data.

Deep learning is an increasingly accessible strategy used in the process of nanomaterial development 3,4. A variety of models and techniques have been developed that attempt to predict supramolecular physicochemical properties to optimize agent design before moving into animal work, from liposomal encapsulation efficiency 5 to metal oxide nanoparticle toxicity 6 to the photonic properties of core-shell nanoparticles 7. Other works push further to outright predict nanomedicine absorption, distribution, metabolism, excretion, and toxicity (ADMET) kinetics based on agent characterization and in vivo delivery kinetic data 8,9. This work, alongside increasingly sophisticated physiologically based pharmacokinetic (PBPK) models, can help direct and provide intentionality to nanomedicine design and its evaluation at an early stage of study, improving the robustness and safety of agents that ultimately transition to clinical trials.

However, preclinical imaging of nanomedicines has only been explored in a limited capacity using machine learning techniques. Kingston *et al.* combined 3D microscopy of optically-cleared tissues with an adaptive learning strategy to automate measurements of nanoparticle distribution, and they subsequently used Support Vector Machine modeling to predict nanoparticle delivery to micrometastases 10. Auto-segmentation models have been developed for use on anatomical CT or MR imaging for whole-body mouse scans 11-13, improving workflows for organ volumetry and metastasis quantification. However, models capable of input of both anatomical (i.e., CT, MRI) and functional (i.e., PET, SPECT) whole-body scans for auto-segmentation and estimation of key pharmacokinetic outputs have not been explored in the field of nanomedicine, despite the immediate and widespread applicability of such tools. Increasing access and investigations of these techniques would provide a clear strategy to optimize and streamline the process of preclinical drug development 14.

**Figure 1:**
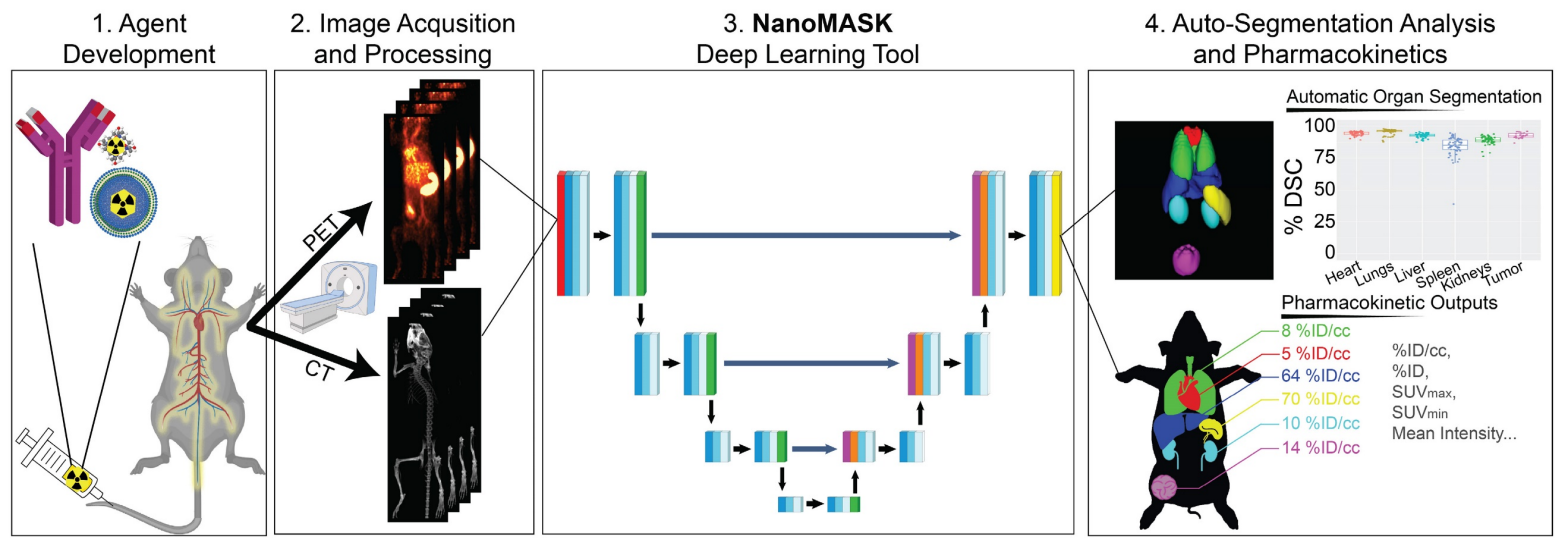
The NanoMASK pipeline streamlines nanomedicine development through automatic analysis of raw anatomical and functional imaging data. It produces high quality, 3-dimensional organ contours and important pharmacokinetic variables such as %ID/cc, organ volume, and SUV_max_.

Here, we explore the application of a 3D U-Net adapted deep neural network to a multifaceted database of longitudinal radioimaging PET/CT whole-body scans of mice, dubbed **NanoMASK** (**Nano**medicine **M**ultimodal **A**I-based **S**egmentation for Pharmaco**K**inetics) (Figure 1). This tool uses a training database containing 355 paired imaging datasets of healthy or 4T1 orthotopic breast tumor-bearing mice acquired up to 48-hour post-injection of a variety of different lipid-shelled microbubbles, agents which exhibit pharmacokinetic profiles similar to lipid nanoparticles 15. Through this work, we demonstrate NanoMASK's ability to generate highly accurate, automated, three-dimensional contours of multiple organ systems relative to the manually contoured ground truth. Furthermore, these machine-generated contours were used to extract important pharmacokinetic measures for the functional imaging data that correlated highly with the values extracted from manual data processing. We explore the dependencies of the NanoMASK model on various dimensions of this dataset, including modality, imaging timepoint, and tumor status, to highlight the importance of training on a nanomaterial-centric dataset with varied functional imaging contrast and its implications for auto-segmentation accuracy. Finally, we validate this model's generalizability through application to external datasets with different nanoparticles, experimental timeframes, and imaging systems. The trained NanoMASK model is freely accessible on Github at https://github.com/bowang-lab/NanoMASK.

## Methods

### Dataset Details

All deep learning techniques were applied to a combined PET/CT dataset generated by the Zheng Lab as part of a comprehensive pharmacokinetic study of a library of custom-formulated, lipid-shelled microbubbles (n=355; 71 mice each measured across 5 timepoints). All animal experiments were conducted in compliance with the guidelines and requirements of the University Health Network Animal Care Committee (AUP 4299, 5922, and 2843.8). Microbubbles made with lipids of chain length varying from 16 to 22 carbons and with inclusion or exclusion of an anionic phosphatidic acid lipid component were formulated with lipid-conjugated porphyrin (pyropheophorbide conjugated to 1-(palmitoyl/stearoyl/behenoyl)-2-hydroxy-sn-glycero-3-phosphocholine, synthesis described in 16). A simple, one-pot chelation strategy was developed that yielded sonication-stable, purification-free association of the microbubbles to ^64^Cu, allowing for quantitative tracking of microbubbles and their subsequent circulating structures across 5 timepoints (1 h, 3.5 h, 6 h, 24 h, 48 h). Studies were conducted in both healthy BALB/c mice and mice bearing orthotopic breast tumors established with a 4T1 murine mammary carcinoma cell line. Following their initial echogenic phase during which they can provide ultrasound contrast, microbubbles transition into smaller, non-echogenic structures and shell fragments with two-phase circulation kinetics possessing a long-phase half-life that varies between 5 and 11 hours, depending on formulation. This pharmacokinetic profile matches well to other supramolecular, PEGylated, lipid-based systems (such as lipid nanoparticles and liposomes) that undergo hepatobiliary clearance 17. Furthermore, the general organ biodistribution patterns of lipid-shelled microbubbles over 48 hours are similar to lipid-based nanoparticles, including predominant uptake within the liver and the spleen and clearance from organs such as the heart, lungs, and kidneys that generally match blood clearance kinetics.

PET/CT acquisitions were conducted using a variety of equipment combinations due to availability (either a combination of (1) Siemens for PET & eXplore Locus Ultra, General Electric for CT; (2) Siemens for PET & X-Rad SmART+ system for CT; or (3) NanoScan, Mediso for combined PET/CT). Co-registration was made possible when utilizing separate equipment for PET/CT through a cross-compatible animal bed. All 355 PET/CT data volumes were quantitatively analyzed through individual, manual contouring of the liver, spleen, kidneys, heart, lungs, and tumor in each image volume using INVEON research workplace software, version 4.2 (IRW; Siemens Healthcare, Ballerup, Denmark). Full-organ, three-dimensional contours were constructed, and methodology was validated through consult with a radiation oncologist (detailed in Appendix A). Voxel intensity data for each organ was exported for processing in Matlab®, version 9.8, R2020b (MathWorks, Natick, Massachusetts, United States). Key pharmacokinetic and biodistribution readouts, including %ID/cc, organ volume, and total organ exposure as represented by the area under curve across the full timeseries (%ID/cc * h), were calculated for each organ using the inscribed segmentations alongside the injected dose decay-corrected to the time of imaging. These are referred to as “ground-truth” in comparison to readouts generated through the auto-segmentation method.

### Data Preparation

To prepare the PET/CT imaging data for processing by the 3D U-Net model architecture underlying NanoMASK, it was necessary to ensure that all data was of consistent format and size. PET and CT datasets, as well as the target organ contours, were re-exported to a common data format (3D NIFTI). All contours were amalgamated into a single file, retaining their identifying index. Accurate co-registration of PET/CT data following re-formatting was ensured by applying the affine transform matrices generated in Inveon Research Workplace using a non-proprietary image analysis software (Simple ITK).

Re-exported and co-registered data was further prepared by cropping the foreground to exclude distal structures such as the head, tail, and animal bed. To compensate for imaging data collected on different machines with different geometries, all CT datasets underwent a global voxel intensity normalization based on the foreground voxel intensities across all training cases. PET datasets were normalized individually by adjusting voxel intensity based on the Z-score (mean subtraction and division by standard deviation) for each 3D image volume. Finally, all data volumes were resampled to conform to the same voxel geometry (0.15 mm x 0.15 mm x 0.80 mm for sagittal, coronal, and axial axes, respectively). These same data preparation steps were applied to the external datasets used to validate NanoMASK's generalizability to other nanomedicines.

### Deep Learning Architecture

NanoMASK uses 3D U-Net 18 as its base network architecture, which contains an encoder and a decoder network. The encoder network aims to extract multiscale image features from the input CT and PET image at different spatial resolutions. The decoder network is used to aggregate the multi-scale information and reconstruct the fine-grained spatial information. Moreover, skip connects are used to bridge the encoder features and decoder features at the same resolution, which can improve the localization precision of target organs. Both the encoder and decoder networks have six resolutions and each resolution has two blocks with convolutional layers, instance normalization 19, and leaky ReLU non-linearity 20. The network input patch size is (64, 160, 160). The first two downsampling operations are only performed on the axes with larger dimensions, resulting in a feature map size of (64, 40, 40). The next three downsampling operations are applied to all the axes, resulting in a feature map size of (8, 5, 5). The last downsampling operation is only performed on the first axis, resulting in the final bottleneck feature maps with a size of (4, 5, 5). The initial number of kernels is 32, which is doubled with each downsampling operation up to a maximum of 320. The downsampling operator in the encoder is implemented as strided convolution while the upsampling operator in decoder is based on transposed convolution. A schematic of this architecture can be seen in Figure S9.

### Training and Testing Protocols

Validation of model accuracy was performed using a 5-fold cross-validation approach. The dataset was randomly split into 5 approximately equal groups. The model was trained a total of 5 times, with each iteration using 4 of the 5 groups (80%) and testing on the remaining 1 group (20%), such that all data volumes participated in the training set during 4 iterations and the testing set for 1 iteration.

The subsetted models designed to evaluate the impact of modality, timepoint, tumor status, and input organ importance were trained using the same training/testing split as the parent NanoMASK model, when possible. The 'PET Only' and 'CT Only' models were trained using an 80/20 split, training on the same 80% of the dataset (using either only the PET or only the CT as input) and tested on the remaining 20% of the combined PET/CT dataset. The '1 h Only', '3.5 h Only', '6 h Only', '24 h Only', and '48 h Only' models were trained on a randomly selected 80% of the data collected at the stated experimental timepoint post-injection of the PET contrast agent and tested on all the remaining data. The 'Healthy Only' and 'Tumor-Bearing Only' models were trained on a randomly chosen 80% of those respective populations within the data and tested on both the remaining 20% of that population and 100% of the other population (the dataset is comprised of roughly ∼35% healthy animals and ∼65% tumor-bearing animals). For the 'Heart Subtracted', 'Lungs Subtracted', 'Kidneys Subtracted', 'Liver Subtracted', 'Spleen Subtracted', and 'Tumor Subtracted' models, the same 80/20 data split was used as the parent NanoMASK model, with the stated restrictions on the input contours provided during training. Details for each models training/testing split can be found in Supplementary Table 2.

### Model Evaluation and Statistical Analysis

The segmentation quality from the deep learning model was assessed by two quantitative measures: Dice similarity coefficient (DSC) and absolute value of relative volume difference (VD). DSC is a widely used metric for evaluating medical image segmentation which measures the region overlap between the 3D segmentation mask from the deep learning model and the ground-truth mask from human experts 21. Volume is an important biomarker for organ quantification and VD measures the volume difference between the segmentation mask and ground-truth mask. Let *G* and *S* denote the segmentation and ground truth, respectively. DSC is defined by


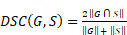

(1)

where the value ranges from 0 (indicating no overlap) to 1 (indicating perfect overlap). VD is defined by


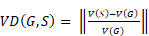

(2)

where *V* (·) is the mask volume. There is no upper bound for the VD score, but the perfect score is 0, indicating consistent volume between *G* and *S*.

The auto-segmentation accuracy of parent NanoMASK model was compared to the experimental subsetted models using one-sided t-tests for the DSC calculated for each organ, utilizing an adjusted significance threshold of *α* = 0*.*05 after Bonferroni correction for multiple comparisons.

The quality of pharmacokinetic predictions of the auto-segmented models was assessed using correlation measures, Bland-Altman plots, and individuals measures of error. Linear models of *y* ∼ *x* were fit for each measure and each model, where y was the value produced by the auto-segmentation model and x was the value produced from the ground-truth manual contours. The Pearson correlation coefficient was calculated for each relationship as a metric for accuracy in prediction. Bland-Altman plots were generated to assess the agreement between auto-segmentation and ground-truth output by plotting the difference of the measures (*y* - *x*) against their average ((*y* + *x*)*/*2) 22. Furthermore, 5 different statistical parameters were used to quantitatively evaluate the difference between model prediction and ground truth, including the mean absolute error (MAE), the root mean squared error (RMSE), the mean absolute relative error (MARE), the root mean squared relative error (RMSRE), and the uncertainty at 95% (U_95_). These are defined as


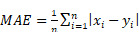

(3)


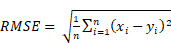

(4)


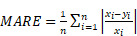

(5)


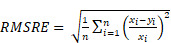

(6)


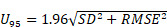

(7)

where *n* is the number of values for a particular measure that are being compared, *x_i_* is the *i*^th^ ground-truth value, and *y_i_* is the *i*^th^ predicted value. All data plotting, significance calculations, and error estimations were performed in R.

In addition, we visualized saliency maps to highlight important regions of an input image that contributed the most to the model's contouring decisions. The saliency maps were generated by gradient-weighted class activation mapping (Grad-CAM) 23, which used the gradients of the predicted class with respect to the feature maps of the last convolutional layer in the model to determine the importance of each feature map. The resulting weights were used to generate a heatmap that highlights the important regions of the input image.

## Results

### NanoMASK Produces Accurate Organ Contours and Pharmacokinetic Predictions

**Figure 2:**
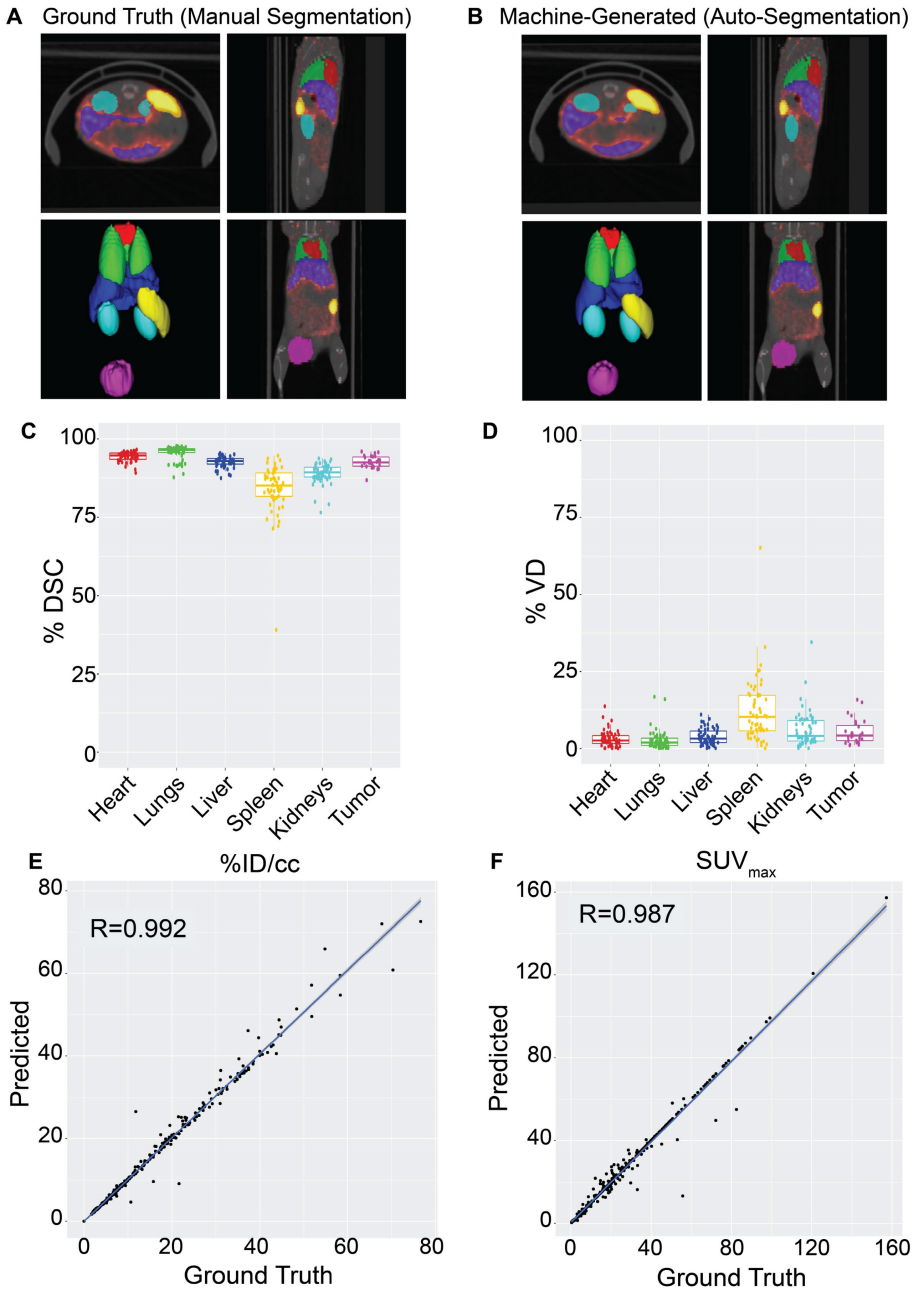
NanoMASK auto-segmentation performance and comparison to manually segmented ground truth. Sample co-registered PET/CT data volume of mouse thorax/abdominal region showcasing multiple views of either **A)** manually-contoured or **B)** machine-generated contours of 3D organ volume segmentations for the heart (red), lungs (green), liver (dark blue), spleen (yellow), kidneys (light blue), and tumor (purple). **C)** Dice similarity coefficient (% DSC) and **D)** percentage volume difference (% VD) for each organ, showing high coherence between manual and machine generated organ volumes. **E)** Percent injected dose per cubic centimeter (%ID/cc) and** F)** maximum standard uptake value (SUV_max_) are two important pharmacokinetic and clinical metrics extracted from machine-generated contours which show a very high correlation to the manually calculated values. Data points in E and F comprise results from all contoured organs collectively.

NanoMASK's auto-segmentation performed very well following 5-fold cross validation. Machine-generated contours were easily visualized alongside the base PET/CT data and appeared virtually indistinguishable when viewed next to the ground-truth contours (Figure 2A,B). When quantitatively assessed, machine-generated contours displayed high spatial overlap with ground-truth contours for all organs tested (Figure 2C,D). This was measured using both the Dice similarity coefficient (% DSC), a widely used spatial overlap index wherein 0% represents no overlap and 100% represents complete overlap, and the percent volume difference (% VD), for which lower values indicate an enclosed volume more similar between the two segmentation methods. The heart, lungs, liver, tumor, and kidneys achieved the highest quality of auto-segmentation (%DSCs of 94.4 ± 1.5%, 95.6 ± 2.3%, 92.6 ± 1.8%, 92.6 ± 2.0 %, and 89.0 ± 3.2%, respectively), while the spleen was modestly lower (84.1 ± 8.1%). The accuracy of the outputted contour volumes was comparable for tested data obtained from different agent compositions, measurement timepoints, and whether animals were healthy or tumor-bearing, despite each of these variables impacting the signal contrast profile of the PET functional data within each imaging volume (Figure S1). Saliency maps, which illustrate the areas most focused upon by the model in making its predictions, suggest an intuitive decision-making framework used by NanoMASK for choosing contoured regions (Figure S2).

Machine-generated contours were capable of reproducing key pharmacokinetic outputs comparable to analysis of the ground-truth data (Figure 2E,F). Metrics of interest that were calculated include the percent injected dose per cubic centimeter (%ID/cc); percent injected dose (%ID); mean, maximum, and minimum standard uptake values (SUV_mean_, SUV_max_, SUV_min_); mean, maximum, and minimum PET voxel intensity; total region volume; and standard deviation of intensity. These values were highly correlated between the ground-truth and machine-generated contours: of particular importance, the %ID/cc, %ID, SUV_max_, and total region volume achieved Pearson correlation coefficients of 0.992, 0.998, 0.987, and 0.996, respectively (others found in Figure S3). Beyond correlation, several other measures of model accuracy, including MARE, MAE, RMSRE, RMSE, and U95, were calculated to compare the quality of NanoMASK predictions to those calculated from manual contours (Supplementary Table 1). These too showed very high prediction accuracy, and they provide the additional benefit of orienting the relative accuracy of NanoMASK with regard to the actual values of the pharmacokinetic metrics being calculated.

### Importance of Modality, Timepoint, Tumor Status, and Input Organs on Quality of Prediction

The relative importance of the different input features used by this auto-segmentation algorithm were evaluated systematically. This was achieved through developing a series of additional auto-segmentation models trained on specific subsets of input data to observe which characteristics result in the greatest drop in quality when removed or subsetted. While this helps to explore the inherent dependencies, strengths, and weaknesses of the NanoMASK model, it also hopes to provide a more general insight as to the necessary qualities of a multimodal preclinical training dataset in order to build a model that outputs high quality contours and accurate pharmacokinetic predictions. Additionally, these tests may indicate which external datasets are most suitable for segmentation using the NanoMASK model, allowing for a more intentional way to apply this model in a generalizable manner.

**Figure 3:**
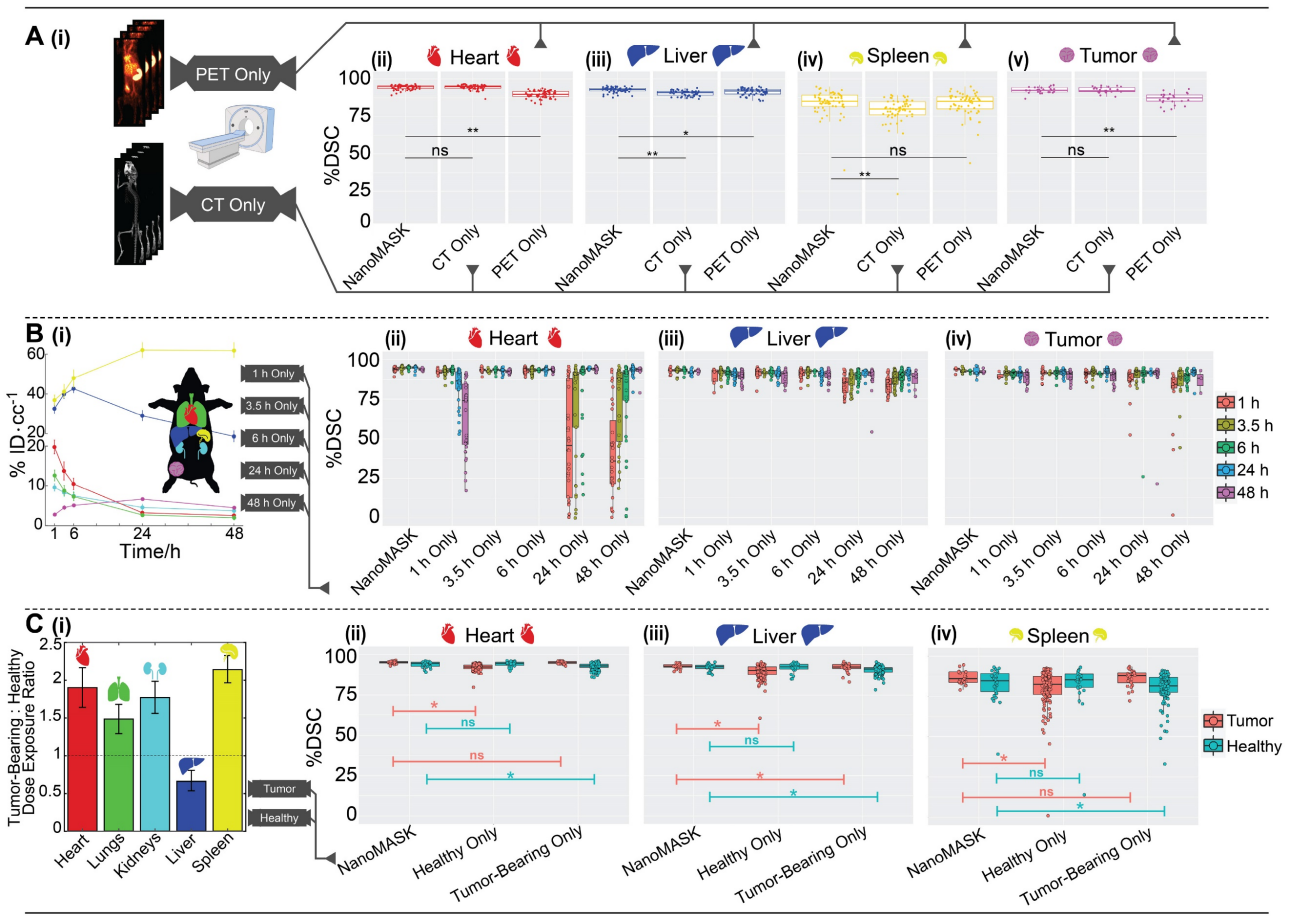
Elucidating the importance of imaging modality, timepoint, and tumor status on auto-segmentation performance through comparison of the parent NanoMASK model to 20+ subsetted models. **A)** CT Only model produced comparable contours to the NanoMASK model for the heart and tumor (p > 0.05), but performed worse for the liver and the spleen (p < 0.005). In contrast, a PET Only model generated less accurate contours for the heart and tumor (p < 0.005), but comparable contours for the spleen (p > 0.05) and liver (p > 0.01). **B)** Sample time series data shows how PET signal can vary over time in each organ. Models trained only on later timepoints (48 h Only) displayed a notable decrease in contouring accuracy when tested on data from earlier timepoints (1 h, 3.5 h) for the heart, lungs, liver, kidneys, and tumor. Additionally, contouring accuracy of the heart was much worse for later timepoints (24 h, 48 h) when created using the model trained only on early timepoints (1 h Only).** C)** Tumor-bearing animals experience differential dose exposure compared to healthy animals. A Healthy Only model showed a decrease of auto-segmentation quality across all organs when tested on tumor-bearing animals (p < 0.05), in tandem with a Tumor-Bearing Only model performing worse on healthy animals. The optimized NanoMASK model outperformed all subset models (A-C), even when tested on their individual training data, illustrating the importance of a diversified, robust training group. * and ** represent significance via a one-sided t-test using an adjusted significance threshold of α = 0.05 or α = 0.005, respectively, after Bonferroni correction for multiple comparisons, while 'ns' means non-significant.

The impact of imaging modality on auto-segmentation accuracy was investigated by training two separate models on solely CT or PET imaging data. While NanoMASK utilizes both PET and CT data as inputs, the exact contribution weight of each modality on the outputted contours cannot be directly parsed. The contribution of PET data is of particular interest, as unlike CT data, it is affected by the injected nanoparticle and changes over time. The auto-segmentation accuracy of these modality-subsetted models compared to the original NanoMASK model (hereafter referred to as the parent model) is shown in Figure 3Ai-iv (additional organs shown in Figure S4Ai-ii). The model trained on only CT data had a slightly reduced contouring accuracy for the liver and the spleen relative to the fully trained model (p < 0.005), but no drop in accuracy was observed for the heart, lungs, kidneys, or tumor (p > 0.05). In contrast, the model trained on only PET data exhibited an opposite trend, showing a decline in contouring accuracy for the heart, lungs, kidneys, and tumor (p < 0.005), but a negligible change in accuracy for the liver and spleen (p > 0.05 and p > 0.01, respectively). Comparison of saliency maps generated by NanoMASK, the PET exclusive model, and the CT exclusive model qualitatively illustrate that the PET exclusive model makes predictions based on organ features more similar to those highlighted by NanoMASK than the CT exclusive model, particularly at later timepoints (Figure S6).

The impact of experimental timepoint on the contouring accuracy and metric output of NanoMASK was evaluated by comparison to five separate subsetted models, each having training data restricted to a single experimental timepoint. Post-injection timepoint is an important imaging parameter because nanoparticles produce vastly different contrast profiles depending on the location of the circulating or extravasated material, with early timepoints (1 h, 3.5 h, 6 h) predominated by a vascular signal that highlights perfusion-dominated organs such as the heart, lungs, and kidneys and late timepoints (24 h, 48 h) emphasizing tissues into which the agent may preferentially accumulate, such as the tumor, liver, and spleen (Figure 3Bi). The results can be seen in Figure 3Bii-iv (additional organs shown in Figure S4Bi-iii), and tables showing measures of significance comparing each subsetted model to the parent NanoMASK model can be seen in Figure S5. Contouring accuracy of the heart experienced the greatest decline using timepoint-subsetted models. The model trained on the earliest timepoint of 1 h performed very poorly when contouring hearts for data collected at 24 h or 48 h post-injection; inversely, the models trained at the later timepoints of 24 h and 48 h experienced a similar decrease in heart contouring accuracy for data collected at 1 h and 3.5 h. Tumor contouring experienced the same trend in accuracy decline as the heart, but of a smaller magnitude. The lungs and kidneys, despite having a similar PET signal profile over time to the heart, only saw a notable decline in accuracy when the model trained on later timepoints (48 h) was tested on earlier data (1 h, 3.5 h). The liver contours experienced no decline in accuracy when using models based on early timepoint (1 h, 3.5 h, 6 h) data, but did have a drop in accuracy for models based on late timepoint (24 h, 48 h) data, specifically for the data collected at 1 h and 3.5 h post-injection. Spleen contouring accuracy was not affected across these different models. Unsurprisingly, these timepoint-sensitivities were validated to be due to differences in functional imaging when trained on models subsetted by both timepoint and image modality (Figure S8). Importantly, the parent model (trained on all timepoints) generated contours with the greatest accuracy relative to the timepoint-specific models. This was true even when using testing data that corresponded to the same timepoint used to train each subsetted model. Overall, these timepoint-specific models show that an auto-segmentation model trained on input data from a diversity of experimental timepoints leads to more robust auto-segmentation predictions across a variety of testing data volumes.

The effect of training on data collected from tumor-bearing mice and training on healthy mice, and vice versa, was tested. While the presence of a tumor can directly affect a nanoparticle's biodistribution in that it serves as a site of preferential uptake, it can also impact off-site nanoparticle biodistribution compared to healthy mice (Figure 3Ci), although the mechanism as to how the immunoreactive, inflammatory state of a tumor-bearing mouse enables this change is controversial 24-27. The comparison between the parent NanoMASK model and two separate models trained on just healthy or tumor-bearing mice can be seen in Figure 3Cii-iv (additional organs shown in Figure S4Ci-ii). Training only on data from healthy animals resulted in a small but significant decrease in contouring accuracy for all organs, with the greatest decreases observed for the liver, spleen, and kidneys. This was due to declining segmentation quality for tumor-bearing animals, with no reduction in quality for the healthy animals. In contrast, the model trained only on data from tumor-bearing animals showed the inverse: a decrease in auto-segmentation quality for the healthy animals, with no drop in quality observed for the tumor-bearing animals. The only organ exception was the liver, which had a drop in quality for both healthy and tumor-bearing animals when using the model trained only on tumor-bearing mice. Overall, this illustrates that auto-segmentation models are sensitive to tumor status. Even when trained on animals of a particular tumor status and tested on that same category of data volumes, the quality of contouring did not exceed that of the parent NanoMASK model, and in fact showed an increase in variance. This test highlights the importance of constructing a model built upon a diverse dataset, including both healthy and tumor-bearing animals, such that it can operate optimally across a variety of test cases.

Finally, organ interdependency was tested using six different models trained on all-minus-one inputted organ contours, including iterations withholding the heart, lungs, kidneys, liver, spleen, and tumor, respectively. From a molecular imaging perspective, PET contrast is derived from the amount of radio-chelated drug present in an area at a particular timepoint; thus, the relative signals within organs of shared biological systems are intrinsically linked to one another via their pharmacokinetic interdependence. Organs that share a common mechanism of drug retention or elimination — such as the mononuclear phagocytic systems within the liver and spleen or the perfusion-dominated signals with the heart, lungs, and kidneys — may provide additional information to the model in unexpected ways. Their relative impact on contouring accuracy and clinical output metrics are summarized in Figure S7. Across all models, there was no reduction in contouring accuracy for any organ given the exclusion of any other input organ from the model. This suggests a high level of independence in segmentation prediction for each organ relative to the other segmentations provided by the model.

### NanoMASK Model Validation Across Multiple Classes of Nanomedicines

NanoMASK performed very well at generating high quality auto-segmentations and accurately outputting key pharmacokinetic variables when applied to in-house PET/CT preclinical data. Furthermore, the algorithms trained on a subset of the multidimensional training dataset illustrated that prediction quality is improved by building a model on data across different timepoints, tumor status, and with input from both modalities. However, to validate the generalizability of this model, it is necessary to test its application on more diverse datasets.

**Figure 4:**
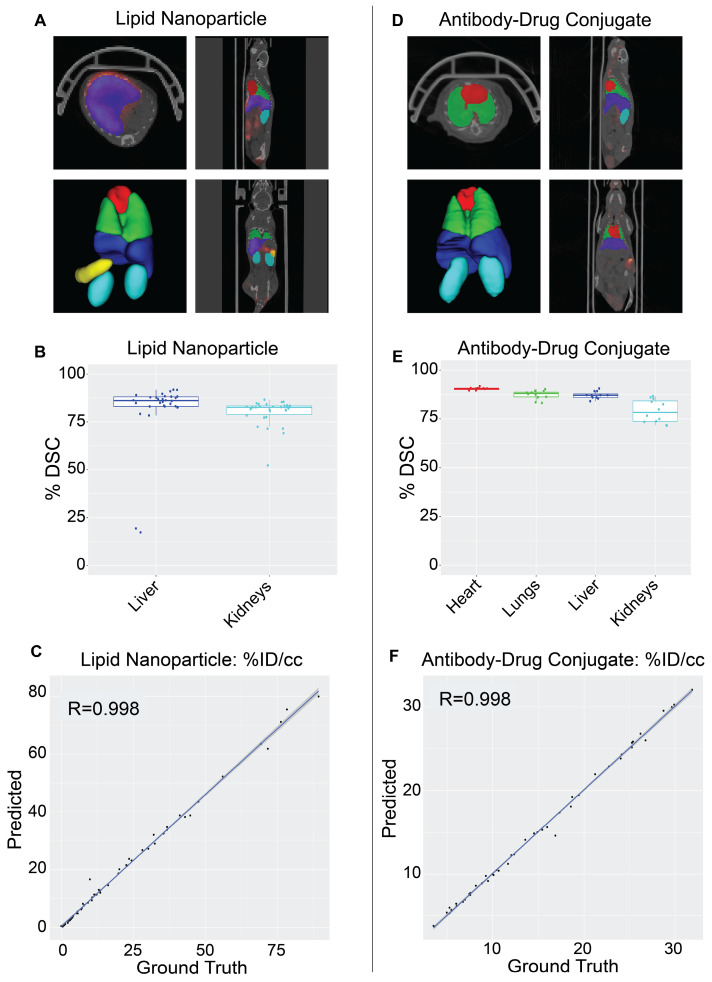
Validation of NanoMASK model on external nanomedicine datasets. NanoMASK generated visually accurate contours for pre-clinical imaging of **A)** lipid nanoparticles and **D)** antibody-drug radioimmunoconjugates. **B)** Volumetric accuracy of NanoMASK compared to manually contoured organs for the liver and kidneys of the lipid nanoparticle dataset **E)** and the heart, lungs, liver, and kidneys of the antibody-drug conjugate dataset, showing a high degree of agreement. **C,F)** The accuracy of the pharmacokinetic output of %ID/cc was shown to be very high for both datasets.

The NanoMASK model was externally validated using two new datasets representing important categories of nanomedicines: (a) a PET/CT dataset of ^64^Cu-chelated porphysomes (n=30), a lipid nanoparticle with a size of 110 nm and which exhibits primarily hepatobiliary clearance (t_1/2_ = 11.1 h 28), and (b) a PET/CT dataset of ^64^Cu-DOTA-panitumumab-F(ab')2 29 (n=12), an antibody-drug conjugate (∼110 kDa) with slow systemic clearance and a nonlinear pharmacokinetic profile due to target-mediated drug disposition 27,30. Both datasets were imaged on different PET and CT instruments than the initial training dataset, and there was no coordination in imaging acquisition parameters. Quantitative evaluation of NanoMASK performance was feasible for all six target organs of the antibody-drug conjugate dataset and the liver and kidneys for the lipid nanoparticle dataset based upon availability of the manual contours (Figure 4).

Both datasets were easily prepared for NanoMASK using a simple data exportation procedure, and co-registration was confirmed visually. Qualitatively, the generated contours for all relevant organ systems were well matched to the 3D data volumes for both datasets. For the lipid nanoparticle dataset, the overlap of auto-segmentations and the ground-truth manual contours for the liver and kidneys were 81.5% and 80.0%, respectively. On inspection of the performance across the different timepoints within the dataset, NanoMASK performed best on data from intermediate timepoints (6 h, 12 h, 24 h) and less optimally at extreme timepoints (3 h, 48 h). These coefficients, while representing a decrease compared to the house-trained testing data, are still reasonably accurate. Importantly, the pharmacokinetic parameters extracted from NanoMASK compared very well to those calculated from the manual contours, showing correlations that exceed 0.997, 0.984, 0.996 for %ID/cc, %ID, SUV_mean_, respectively. Thus, while there is a moderate drop in volumetric accuracy when tested in a new dataset, the extracted clinical metrics remain highly accurate. For the antibody-drug conjugate, the DSC for the heart, lungs, liver, and kidneys were 90.4%, 87.3%, 87.2%, and 78.9%, respectively. There were no observed performance differences across the different timepoints (6 h, 24 h, 48 h) in the dataset. This represents an even higher accuracy than the lipid nanoparticle data, showing that it is highly generalizable to different nanostructures if the form of the data is suitable for input into the model. However, NanoMASK was not able to generate sufficiently accurate contours for the spleen (likely due to differences in CT contrast) or the tumor (likely due to a different subcutaneous location and a 10-fold size difference). The pharmacokinetic parameters extracted for the antibody-drug conjugate data also matched very well to those from manual contours, with correlations of 0.998, 0.996, and 0.986 for %ID/cc, %ID, and SUV_max_, respectively.

Overall, these test cases showcase how NanoMASK can be easily and generally applied to generate informative, 3-dimensional auto-segmentations for key organ systems and extract critical pharmacokinetic data that is almost indistinguishable from that which was calculated through the more time-intensive, manual contouring procedure.

## Discussion

### A Readily-Applicable Auto-Segmentation Model for Multimodal Preclinical Data

In this study, we introduced NanoMASK, a 3D U-Net-based deep-learning tool capable of highly accurate, 3-dimensional organ auto-segmentation for PET/CT multimodal imaging data in mice. For an automated tool to suitably serve this purpose, it would need to be robustly trained to work across a variety of image settings, provide contouring for many organs of pharmacokinetic interest, work rapidly in an unsupervised fashion, and match or exceed the accuracy of manual contours constructed with input from a nuclear imaging expert. NanoMASK meets all these criteria. It was trained using 355 input PET/CT data volumes, the largest training dataset for a preclinical auto-segmentation project that the authors can determine. This inclusion of data across different agent formulations, experimental timepoints, animal tumor status, and PET/CT instruments and settings was explicitly shown to enable greater generalizability to test data than models trained on fewer, less diverse datasets. NanoMASK can provide contours for six major organs of interest that comprise key systems related to agent circulation, processing, and excretion, including orthotopic breast tumors. It can generate contours in less than a minute without any manual input beyond the base PET/CT imaging data, and the thousands of produced contours were shown to be highly accurate across several measures of volumetric comparison. NanoMASK's base code and the full model are publicly available for immediate and rapid application to any user's own dataset. We hope to continue to improve the accuracy and usability of this model as we incorporate more varied data into our training set and reframe the model using SAUNet, an architecture optimized for interpretability 31.

The ability to operate on multimodal data and automatically produce key pharmacokinetic readouts is a unique feature of NanoMASK that sets it apart from currently available preclinical auto-segmentation models. This interpretation of functional imaging data is often the primary desired result of preclinical imaging in drug development, and its direct incorporation into this model's operation improves its utility and further helps this tool streamline the analysis of in vivo work. Parameters such as %ID/cc, %ID, SUV_max_, and mean PET intensity are shown to be extremely accurate across thousands of comparisons to manually calculated values. For instance, MAREs for the heart, lungs, kidneys, liver, spleen, and tumor for the %ID/cc were all below 0.2%, a prediction accuracy that easily surpasses that of interoperator accuracy comparisons 11,32. Additional pharmacokinetic calculations that utilize functional imaging intensity and experimental data, such as organ residence time or radiation equivalent dose, could easily be incorporated into the model outputs to suit the primary measures of a particular study.

When applied to new datasets, NanoMASK continued to produce highly accurate contours. This included testing on radio-imaging studies of lipid nanoparticles and antibody-drug conjugates, which represent two of the most widely used classes of nanomedicines in both preclinical development and clinical application. Furthermore, these two drug classes possess different pharmacokinetic profiles, and thus they give different contrast profiles over time to organs of circulation (heart, lungs) as well as organs of clearance (liver, spleen, kidneys) and sites of uptake (tumor, healthy tissue). NanoMASK's success in handling this data suggests that it is likely generalizable to other varieties of nanomedicine that can be evaluated using a PET/CT platform 33,34, including radio-functionalized inorganic nanoconstructs such as mesoporous silica nanoparticles 35, gold nanoparticles 36,37, superparamagnetic iron oxide nanoparticles 38, and quantum dots 39; alternate lipid structures such as lipoprotein-like nanoparticles 40, microbubbles 41, and nanodroplets 42; and polymer-based nanostructures such as nanospheres 43 and dendrimers 44. These agents, which often undergo a significant course of preclinical optimization to assess the pharmacokinetic impact of changes to formulation and dosage, are ideal candidates for input to this model, which poses to massively expedite the process of image volume analysis. While not explicitly trained and tested on molecular PET contrast agents, it would be a future area of interest to see if NanoMASK can operate well on agents beyond the nano-paradigm.

### Fundamental Lessons about Multimodal Auto-Segmentation Models Learned from NanoMASK Subsetted Experiments

With continuing breakthroughs in model architecture and potentials for personal adaptation to ideally suit a particular dataset, this work acknowledges that further improvements to NanoMASK's model architecture and usability are inevitable. Thus, several additional tests were performed to probe more fundamental concepts related to the quality and diversity of training data used to build a U-Net-based auto-segmentation model in the hopes of assisting others wishing to construct similar models optimized for their experimental pipeline. In this, we discovered several key factors that we believe to be broadly generalizable principles for multimodal image analysis.

First, all the auto-segmentation models that were constructed on subsetted datasets along a particular dimension — timepoint, modality, tumor status, or input organ — failed to outperform the parent NanoMASK model in terms of contouring accuracy, even when tested only on the same experimental subset used to train the model. This suggests that broader training datasets are ideal for model construction, even if the model's intended application only represents a subset of the training data.

Second, using functional imaging (PET, in this case) in combination with typical anatomical imaging (CT, in this case) improved overall auto-segmentation outcomes. Given the variability of functional imaging across timepoint and agent formulation, it was not hypothesized to consistently improve auto-segmentation quality, but this work shows it provides modest improvements in volumetric accuracy and pharmacokinetic predictions. Furthermore, even the subsetted model trained on only the functional imaging performed unexpectedly well, outperforming the model trained purely on anatomical imaging with regards to outputs for organs exhibiting high functional imaging contrast (liver and spleen). This tracks intuitively with the fact that these two organs represent the majority of signal derived from the nanoparticle dataset, as these agents showcase a highly hepatic and splenic mode of processing and clearance typical to nanomaterials. While abandoning anatomical imaging is not advised, this illustrates that well-trained models are powerful tools that can generate contours on data which would be impossible to contour manually.

Third, diversity in experimental timepoints of training data was found to be incredibly important to maintain auto-segmentation accuracy in tested data. All organs (excluding the spleen) were contoured more poorly when using a model trained on an early timepoint and tested on later timepoints, and vice versa. This suggests that any auto-segmentation model that is to be applied to pre-clinical data across a diversity of experimental timepoints should be trained on data that spans those experimental timepoints.

Fourth, tumor status was a significant factor for auto-segmentation volumetric accuracy. Preclinical work across multiple classes of nanoparticles have shown that tumor burden can alter the pharmacokinetic profile of an agent, such as through changes in sites of active uptake 27 or cancer-induced physiological changes such as increased splenic activity 26. If volumetric accuracy is the goal of auto-segmentation, the model used should be trained on both healthy and diseased animal phenotypes. Furthermore, to obtain accurate tumor contouring and classification, models should be trained using tumor locations similar to those of the test dataset.

Finally, there was no measurable interdependency between the different organs NanoMASK was trained to output. This suggests that models built using a 3D U-Net architecture can be readily modified to predict auto-segmentations for more (or less) organs without expecting any change in overall accuracy. This may include auto-segmentation functionality for other important tissues such as the bone marrow (site of immunomodulation), the brain (a key negative control), or the bladder (a site of rapid excretion for smaller sized therapeutics).

## Conclusion

In this work, we introduced NanoMASK, the first auto-segmentation tool developed specifically for applications in nanomedicine. It combines both anatomical and functional imaging data to produce high quality contours of key organ systems related to agent pharmacokinetics and biodistribution. It was shown to be highly robust across different qualities of input data and generalizable to several nanomedicine classes. Importantly, it can generate pharmacokinetic outputs automatically with extremely high accuracy relative to manually calculated data. This poses to dramatically reduce the time and expertise required to utilize nanomedicine preclinical imaging data to its fullest potential. It is our hope that open-access usage of this model or its principal architecture will integrate easily into the preclinical pipeline for nanomedicine platform optimization and expedite its more laborious aspects.

## Supplementary Material

Supplementary figures and tables.

## Abbreviations

3D: three-dimensional
ADMET: absorption, distribution, metabolism, excretion, and toxicity
AUP: animal use protocol
cc: cubic centimeter
CT: computed tomography
Cu (^64^Cu): copper/copper-chelated
DOTA: tetraazacyclododecane-1,4,7,10-tetraacetic acid
DSC: Dice similarity coefficient
Grad-CAM: gradient-weighted class activation mapping
h/hr: hour
ID (%ID, %ID/cc): injected dose (percentage of injected dose, percentage of injected dose per cubic centimeter)
ITK: Insight Toolkit
MAE: mean absolute error
MARE: mean absolute relative error
MR(I): magnetic resonance (imaging)
n: number of experimental replicates
NanoMASK: Nanomedicine Multimodal AI-based Segmentation for Pharmacokinetics
PBPK: physiologically-based pharmacokinetic
PET: positron emission tomography
PEGylated: containing polyethylene glycol
ReLU: rectified linear unit
ROI: region-of-insert
RMSE: root mean squared error
RMSRE: root mean squared relative error
s: second
SD: standard deviation
SmART: Small Animal Radiation Therapy
SPECT: single-photon emission computer tomography
STTARR: Spatio-Temporal Targeting and Amplification of Radiation Response
SUV (SUV_max_, SUV_mean_, SUV_min_): standard uptake value (maximum, mean, minimum)
U95: uncertainty at 95%
VD: volume difference

## Appendix A: Full Organ, Three-Dimensional Contouring Protocols

The training data for NanoMASK comprised of 355 PET/CT volumes, all of which were manually contoured to identify functional contrast within the heart, lungs, liver, spleen, kidneys, and tumor. Volumes were constructed to realistically cover the full organs rather than a simpler volumetric volume-of-interest that may not fully represent the entire organ, an important consideration for accuracy given the heterogeneous, asymmetrical nature of these organs. The techniques utilized were informed through consultation with a nuclear imaging expert. The procedure was guided in part by the co-registered CT data. All contours involved drawing separates 2D areas across multiple slices of the anatomical plane and interpolating across them to generate volumes representative of the contoured area. After their individual construction, the different organ contours were evaluated together to ensure no overlap. Full instructions for the protocols used to contour each organ are detailed below.

*Heart/Lungs* — The entire thoracic cavity was contoured across the sagittal plane using the rib cage as a guide. This volume was thresholded using the CT intensity data to provide a rough estimate of the lungs within the thorax, given their echolucency on CT. This approximation of the lungs was manually adjusted to ensure inclusion of the less echolucent bronchi, bronchioles, and pleura. An initial estimate of the contour of the heart was generated based on the differential volume between the thorax and lung contours. This was manually adjusted to ensure that the primary vessels of the lungs, mediastinum, and other portions of the thorax were not included within the heart contour. The aortic arch was included within the contour of the heart, but not the portions of the vessels ascending cranially beyond this point.

*Liver* — The liver was contoured along the axial plane. The top section of the liver was contoured moving caudally from the dome of the liver as it abuts the diaphragm. As the different lobes of the liver descend different distances caudally in the abdomen and break up the projection of the liver in the axial plane, these were contoured separately and joined into one final volume. Special consideration was taken to avoid overlap with other abdominal structures such as the stomach, spleen, and intestines, as well as retroperitoneal structures including the kidneys, aorta, inferior vena cava, pancreas, and duodenum. This task aided by a combination of guidance from CT and PET data, the latter of which was notably brighter in the liver than those other structures, with the exception of the spleen.

*Spleen* — The spleen was contoured along the axial plane, starting at the apex of the diaphragmatic surface and proceeding caudally. The tasks was aided by a combination of guidance from CT and PET data; particularly, the contrast between the exceptionally low signal in abutting structures (diaphragm, stomach) and the high signal of the spleen helped to clearly define its boundaries. Reference to the CT data helped to prevent miscountouring as a result of spillover of signal from the PET imaging. The splenic veins and arteries, when discernibly separate from splenic hilum, were not contoured as part of the spleen.

*Kidneys* — The kidneys were contoured individually along the coronal plane. Their retroperitoneal location helped to distinguish and separate them from nearby abdominal structures. Contouring was mainly guided by CT data given the variability in PET signal over time, which could lead to inconsistent volumes. Starting dorsally, the region was contoured from the posterior to the anterior surface, taking care to avoid overlap between both the apical portion of the right kidney and the liver and the apical/medial portion of the left kidney and the spleen. The renal veins and arteries, when discernably separate from the body of the renal hilum, were not contoured as part of the kidneys. Even though they were contoured separately, they were considered together when expressing aggregate measures of the PET data.

*Tumor* — Tumor contours were created across either the sagittal or coronal plane and then subsequently refined across axial, sagittal and coronal planes. Tumors were located in the right 5th (inguinal) mammary fat pad. The tumor boundaries were delineated from surrounding healthy tissue dorsally using a combination of PET and CT data cues. Surrounding fascia and fat pad appeared more consistently hypodense on CT relative to the less consistent, “patchy“ hyperdense areas of the tumor. At times, clear capsule-like boundaries could be seen that separated tumor from surrounding hyperdense abdominal tissue. However, for the majority of tumors, evaluation of patterns of regional hyperdense tissue growth over the 1 to 48 hour timeframe during which tumor growth was expected was needed to clearly delineate tumor boundaries from surrounding hyperdense abdominal tissue. The bright PET signal that emanated from arteries lateral (i.e., femoral and proximal caudal femoral arteries) or medial (i.e., external pudendal artery) at earlier timepoints was also used to better delineate tumor boundaries (especially the lateral boundary) by monitoring areas of bright linear signal that decreased over time. Additionally, hypervascular tumor tissue often displays a brighter PET signal than the surrounding tissue at later time points (24 and 48 hours), although authors were careful not to rely on this as a means of delineating tumor boundaries due to potential bias it may pose between different formulations exhibiting different degrees of tumor uptake.
