# Supplementary material for: Deep learning for automatic organ and tumor segmentation in nanomedicine pharmacokinetics
Source: Theranostics. 2024 Jan 1;14(3):973–87. doi: 10.7150/thno.90246 (PMC10797295; doi:10.7150/thno.90246)
Supplement: Supplementary file 1 — Supplementary figures and tables. [file thnov14p0973s1.pdf]

Supplementary Information

Supplementary Figures

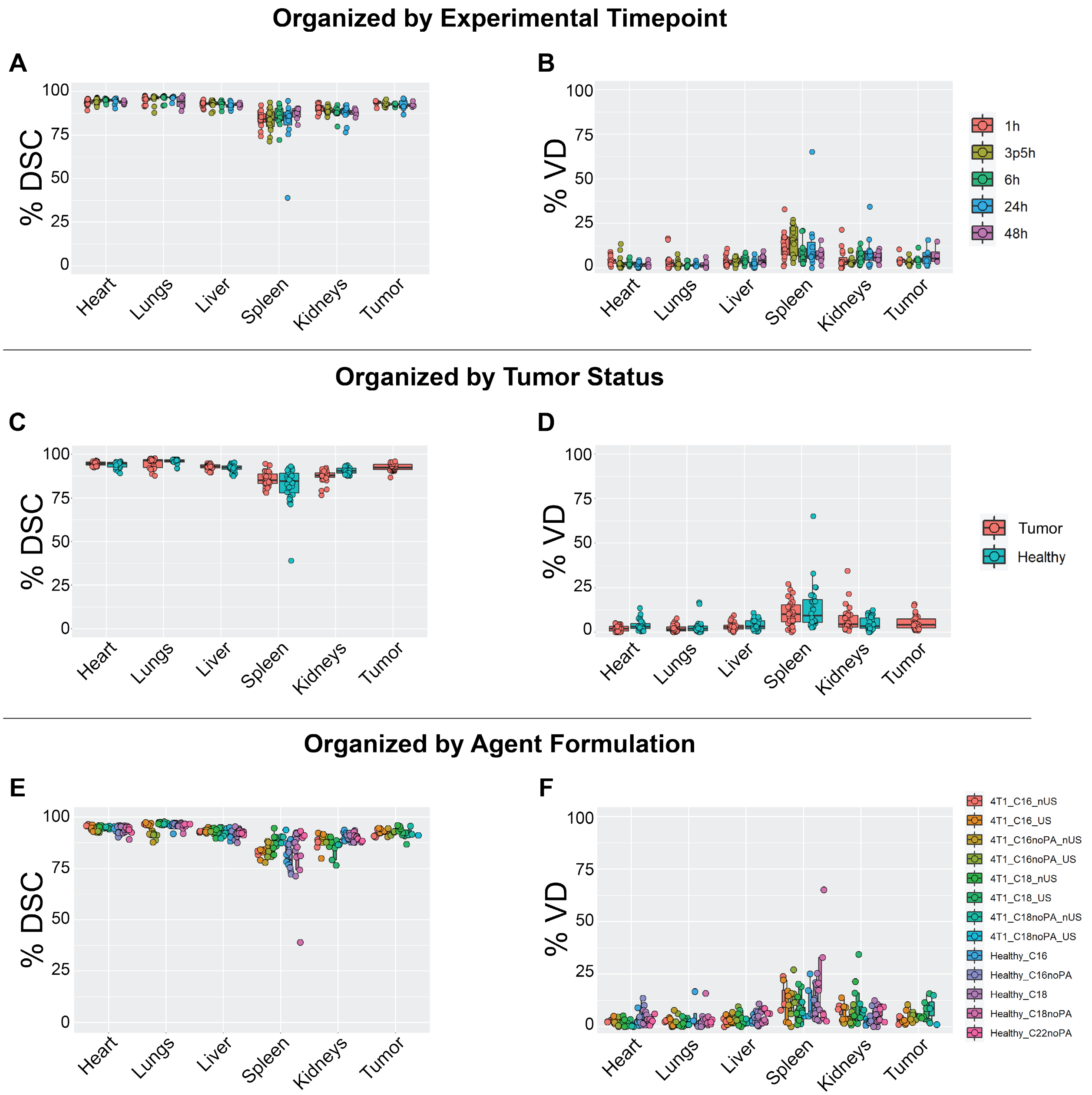

Figure S1: Quantitative volumetric comparison between NanoMASK and manually-created organ contours with data separately plotted according to sub-classifications of the tested dataset: experimental imaging timepoint (**A,B**), tumor status (**C,D**), or nanoparticle formulation (**E,F**).

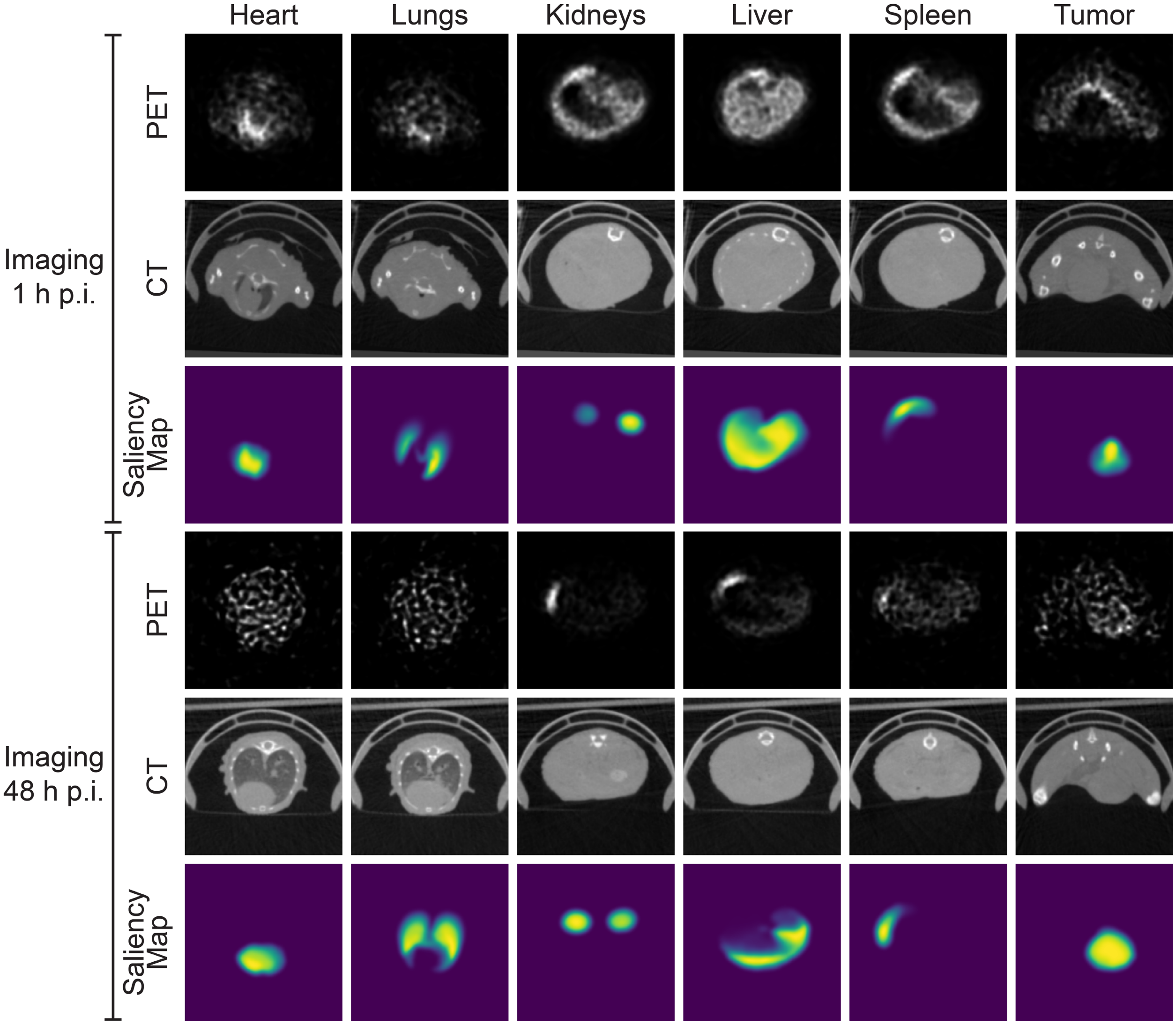

Figure S2: Saliency maps produced by NanoMASK in comparison to selected views of PET and CT data. While PET data clearly varies between early and late timepoints for most organs, CT anatomical imaging remains consistent. Saliency maps show logical identification of features for organs across timepoints.

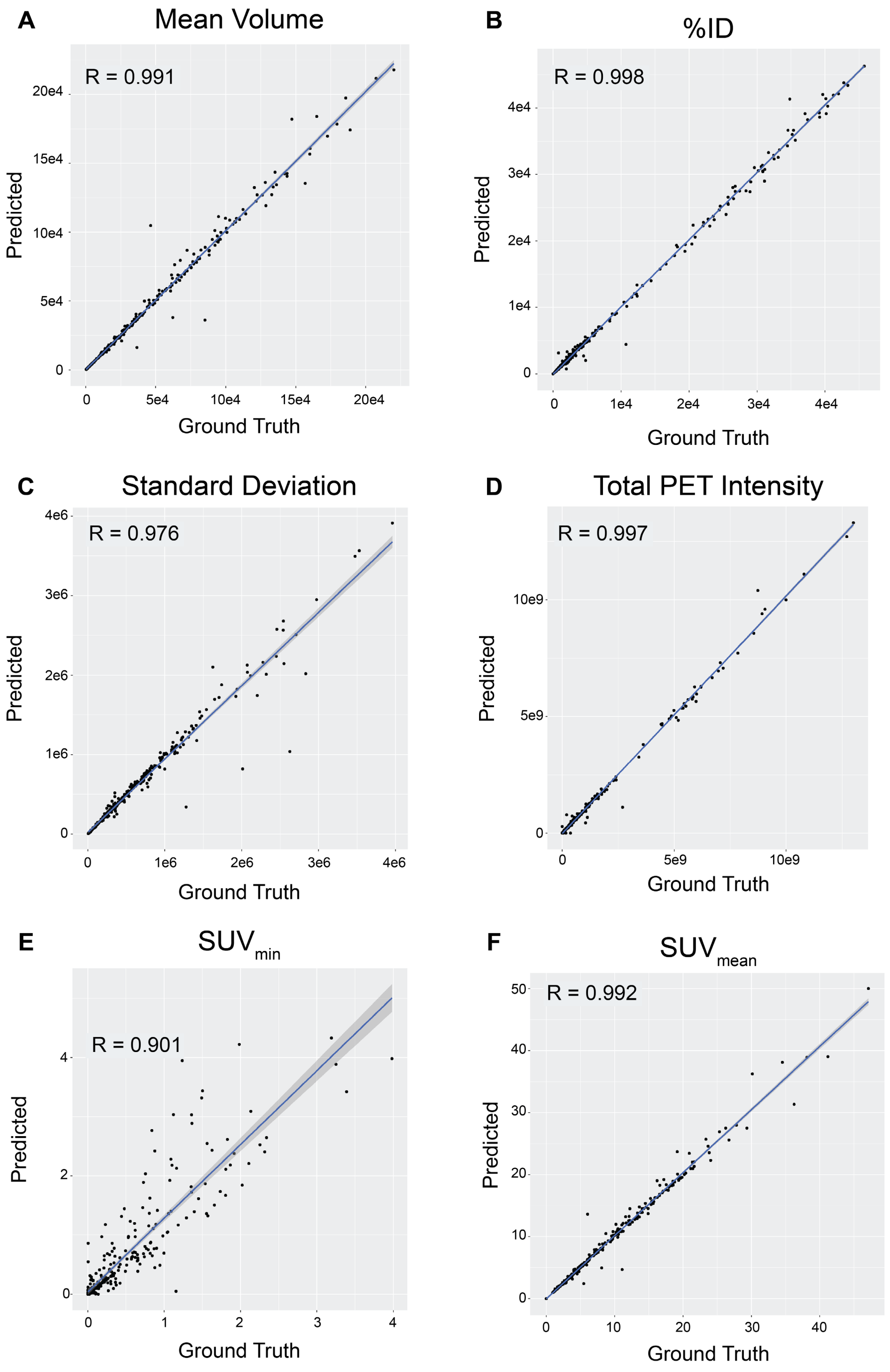

Figure S3: Additional correlations between ground-truth and predicted values for important pharmacokinetic variables. **A)** Mean volume, **B)** percent of injected dose (% ID), **C)** standard deviation of PET voxels in contoured volume, **D)** total PET intensity of voxels in contoured volume, **E)** minimum standard uptake value (SUV_{min}), and **F)** mean standard uptake value (SUV_{mean}) all show reasonably high correlation, showing that NanoMASK functions well to generate any of these important pharmacokinetic variables.

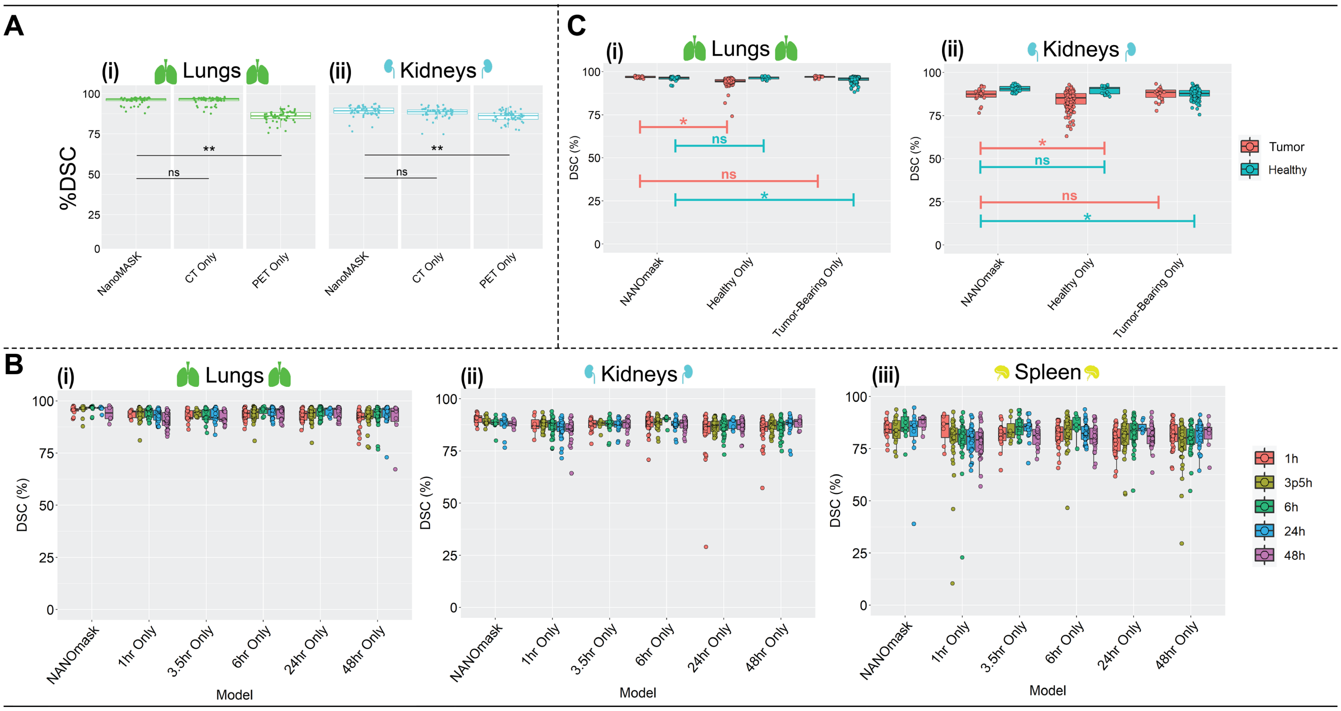

Figure S4: Additional organ comparisons between NanoMASK and 20+ additional models subsetted according to imaging modality, timepoint, and tumor status as a means of investigating their impact on auto-segmentation performance. **A)** Lungs and kidneys are contoured equally well comparing NanoMASK to a CT Only model ($p > 0.05$) but a reduction in performance quality is seen compared to a PET Only model ($p < 0.05$), a trend which matches that of the heart, another organ with variable PET signal over 48 h as drugs are cleared from circulation. **B)** Similar to the heart, lung and kidney contouring accuracy was reduced when using a model trained only on later timepoints (48 h Only) and tested on data from earlier timepoints (1 h, 3.5 h); however, unlike the heart, it did not show the reciprocal trend of poor contouring of late-timepoint data when trained exclusively on early-timepoint data. The spleen showed no change in contouring accuracy across any timepoint-subsetted models. **C)** Contouring accuracy of lungs and kidneys is reduced when tumor-bearing animals are contoured using a Healthy Only model ($p < 0.05$) and when healthy animals are contoured using a Tumor-Bearing Only model ($p < 0.05$), similar to all other contoured organs. NanoMASK either outperformed or matched all subset models (A-C) in contouring accuracy.

Heart

	NanoMASK vs 1hr Only	NanoMASK vs 3.5hr Only	NanoMASK vs 6hr Only	NanoMASK vs 24hr Only	NanoMASK vs 48hr Only
1hr	ns	ns	ns	*	*
3.5hr	*	*	*	*	*
6hr	*	*	*	*	*
24hr	*	*	ns	ns	ns
48hr	*	*	ns	ns	ns

Lungs

	NanoMASK vs 1hr Only	NanoMASK vs 3.5hr Only	NanoMASK vs 6hr Only	NanoMASK vs 24hr Only	NanoMASK vs 48hr Only
1hr	ns	*	*	*	*
3.5hr	*	ns	*	*	*
6hr	*	*	ns	*	*
24hr	*	*	*	ns	*
48hr	*	ns	ns	ns	ns

Liver

	NanoMASK vs 1hr Only	NanoMASK vs 3.5hr Only	NanoMASK vs 6hr Only	NanoMASK vs 24hr Only	NanoMASK vs 48hr Only
1hr	ns	*	*	*	*
3.5hr	ns	ns	ns	*	*
6hr	*	ns	ns	*	*
24hr	*	ns	ns	ns	ns
48hr	*	*	*	*	*

Spleen

	NanoMASK vs 1hr Only	NanoMASK vs 3.5hr Only	NanoMASK vs 6hr Only	NanoMASK vs 24hr Only	NanoMASK vs 48hr Only
1hr	ns	ns	ns	*	ns
3.5hr	*	ns	ns	ns	*
6hr	*	ns	ns	ns	*
24hr	ns	ns	ns	ns	ns
48hr	*	*	*	*	ns

Kidneys

	NanoMASK vs 1hr Only	NanoMASK vs 3.5hr Only	NanoMASK vs 6hr Only	NanoMASK vs 24hr Only	NanoMASK vs 48hr Only
1hr	ns	*	*	*	*
3.5hr	*	ns	ns	*	*
6hr	ns	ns	ns	*	ns
24hr	ns	ns	ns	ns	ns
48hr	*	ns	ns	ns	ns

Tumor

	NanoMASK vs 1hr Only	NanoMASK vs 3.5hr Only	NanoMASK vs 6hr Only	NanoMASK vs 24hr Only	NanoMASK vs 48hr Only
1hr	*	*	*	*	*
3.5hr	*	ns	ns	*	*
6hr	*	ns	ns	ns	*
24hr	ns	ns	ns	ns	ns
48hr	*	*	*	ns	ns

Figure S5: Illustration of statistical tests (one-sided t-tests) comparing NanoMASK to timepoint-subsetted auto-segmentation models for each organ. Data was split based upon experimental timepoint and compared separately. * represents significance using an adjusted significance threshold of $\alpha = 0.05$ after Bonferroni correction for multiple comparisons (n=25), while 'ns' means non-significant.

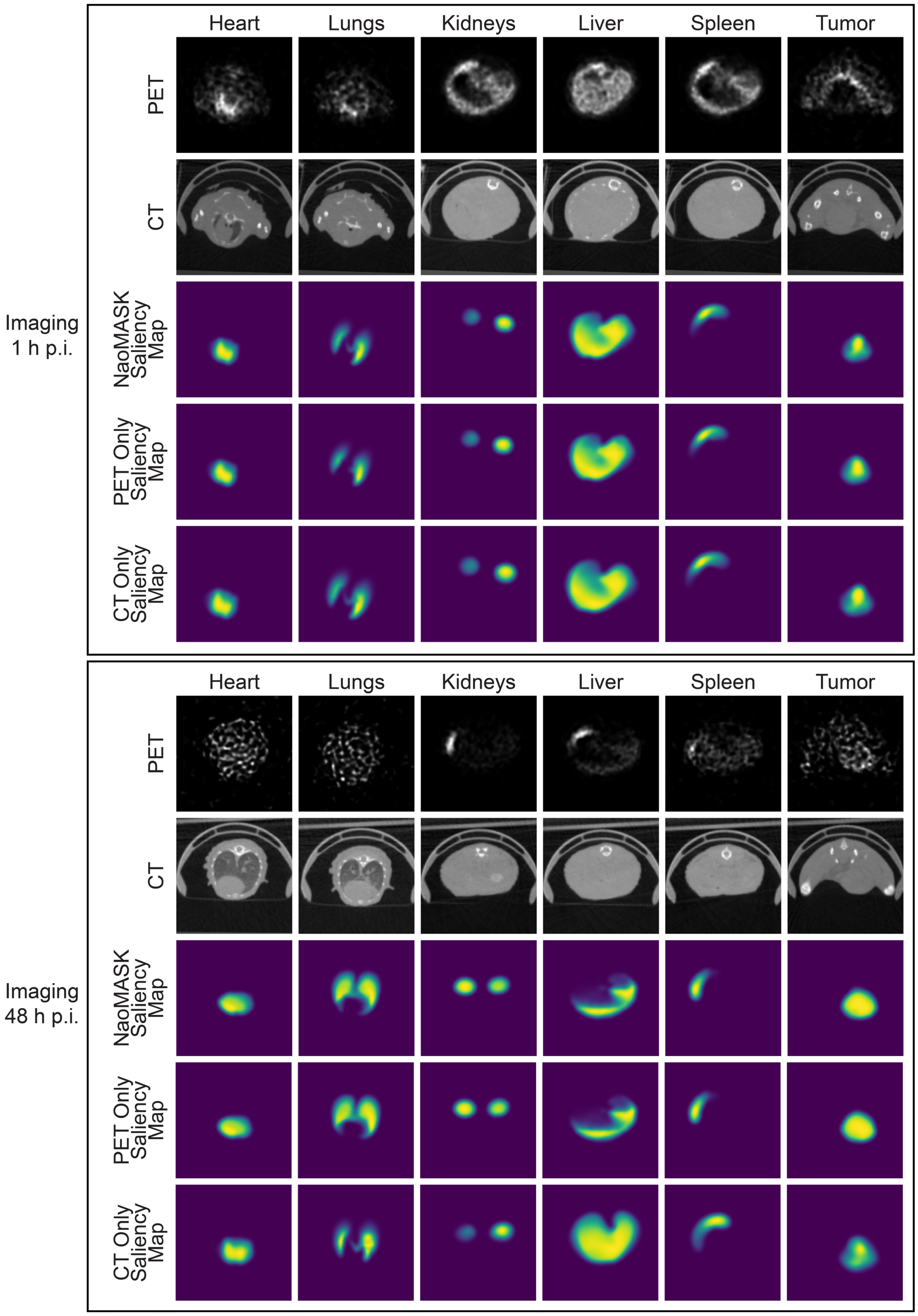

Figure S6: Comparison of saliency maps produced by NanoMASK in models trained exclusively on PET or CT data. Maps show more similar confidence in contouring specific regions when compared to the PET-trained model rather than the CT trained model, despite this not directly correlating with overall contouring accuracy. The variety in functional imaging contrast clearly informs contouring decisions made by NanoMASK.

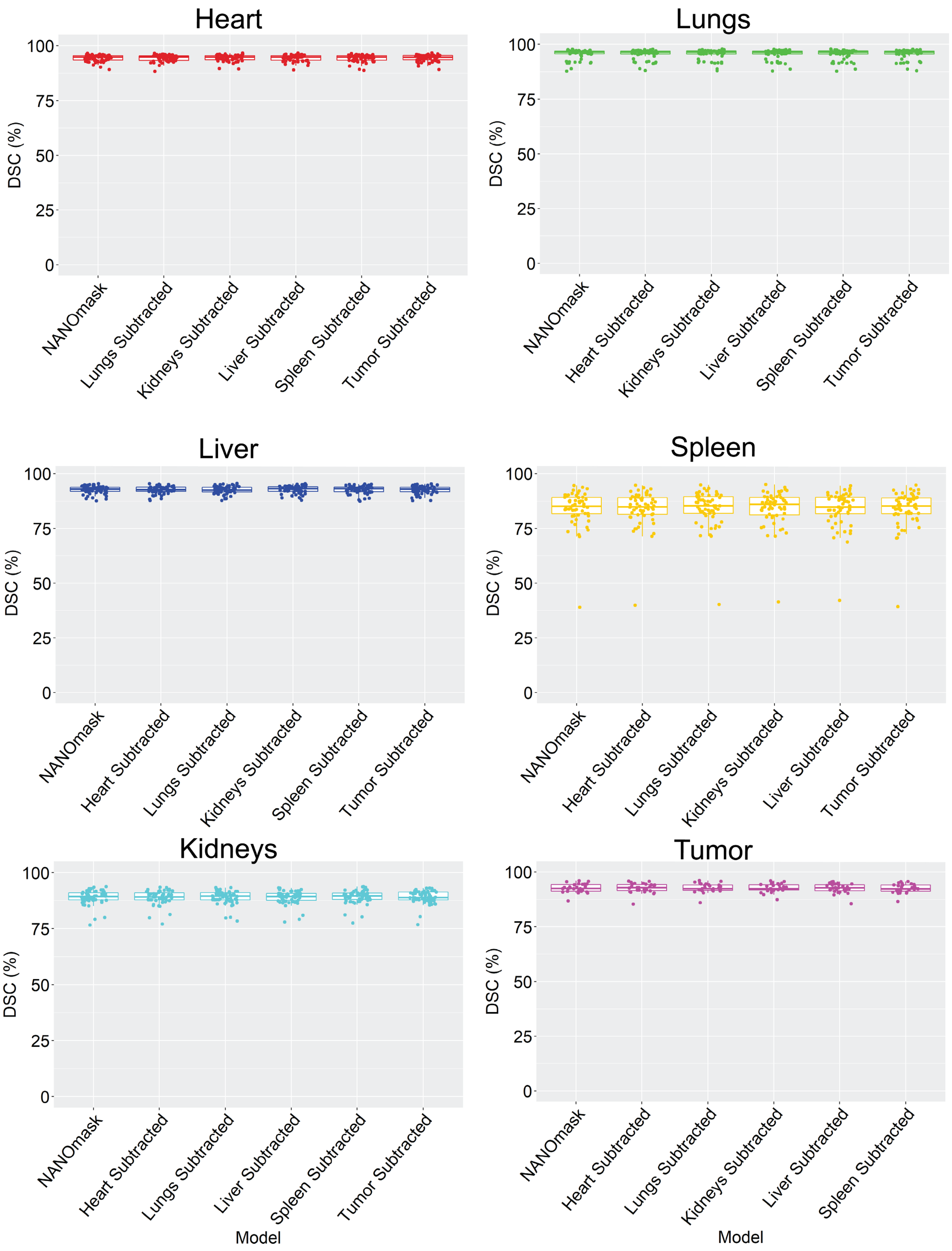

Figure S7: Experimental comparison to illustrate auto-segmentation dependency on the manual organ contours providing as training input for the auto-segmentation model. Six additional auto-segmentation models were trained using the same training/testing data split architecture as the optimized model (NanoMASK), but selectively omitting each organ contours as a training input and examining its effect on the contouring accuracy of the other organs predicted by the model. The auto-segmentation quality for all of the subtracted models were not significantly different from the optimized model for any organ, suggesting that the organ inputs function highly independently from one another in the auto-segmentation model.

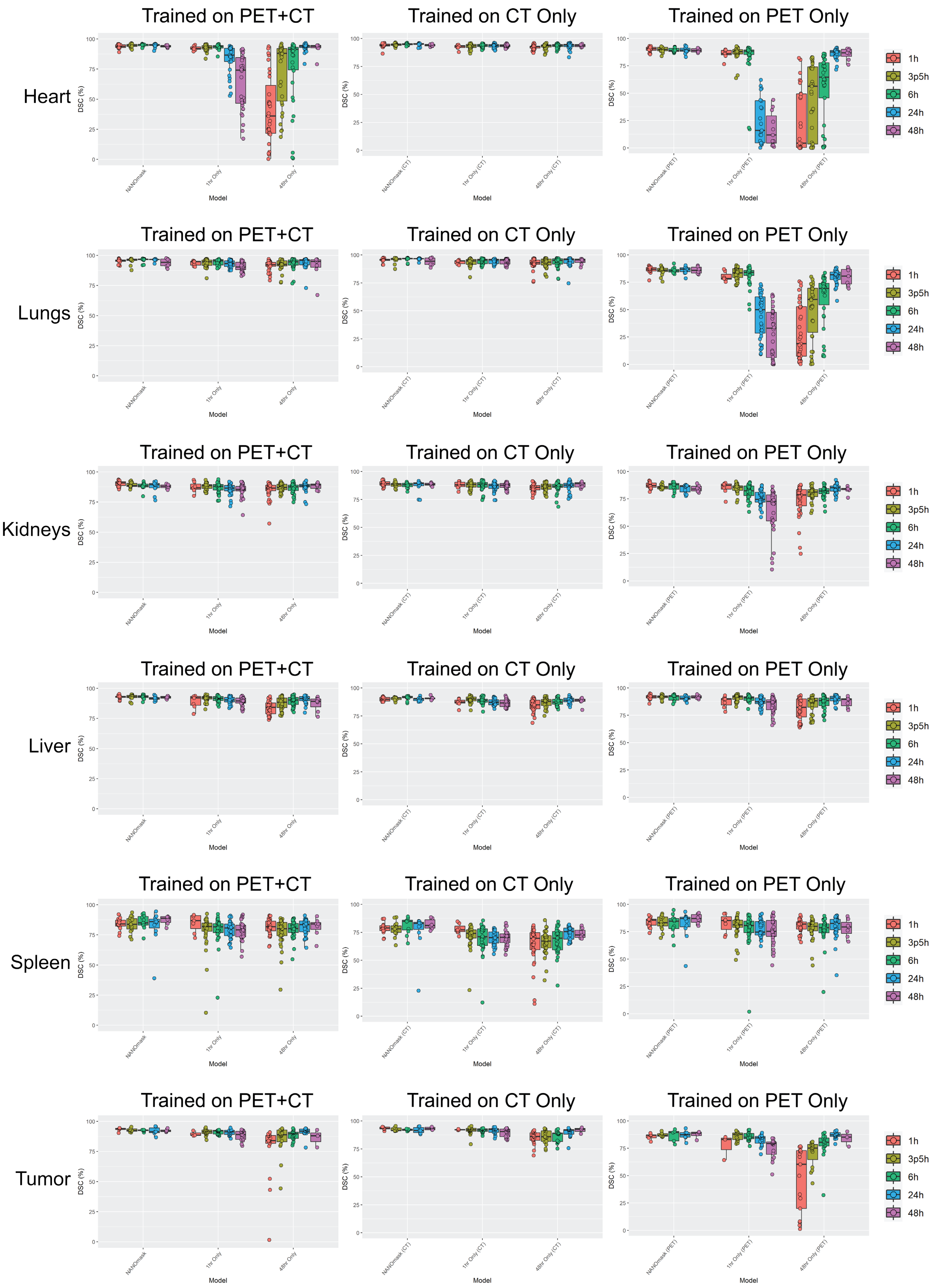
Trained on PET+CT
Trained on CT Only
Trained on PET Only
Heart
Lungs
Kidneys
Liver
Spleen
Tumor
DSC (%)
Model
NANOmask
1hr Only
48hr Only
NANOmask (CT)
1hr Only (CT)
48hr Only (CT)
NANOmask (PET)
1hr Only (PET)
48hr Only (PET)
1h
3p5h
6h
24h
48h

Figure S8: Experimental comparison to prove that timepoint-subsetted auto-segmentation models produce poor segmentations due to the contribution of the functional imaging input. In addition to the NanoMASK parent models and the 5 timepoint subsetted models trained only on data from a single experimental time, 4 additional auto-segmentation models were created: (1) using only 1 h data and only CT information as an input, (2) using only 1 h data and only PET information as an input, (3) using only 48 h data and only CT information as an input, and (4) using only 48 h data and only PET information as an input. Each row represents volumetric comparison for a particular organ (from top to bottom: heart, lungs, kidneys, liver, spleen, tumor). Each column represents the modalities on which the models were trained (from left to right: trained on both PET and CT data, trained on CT data only, trained on PET data only). It is clear that the drastic reduction in quality when a timepoint-subsetted model is applied to untrained timepoints is massively impacted in the PET-trained models, while the CT-trained models experience less decline in accuracy.

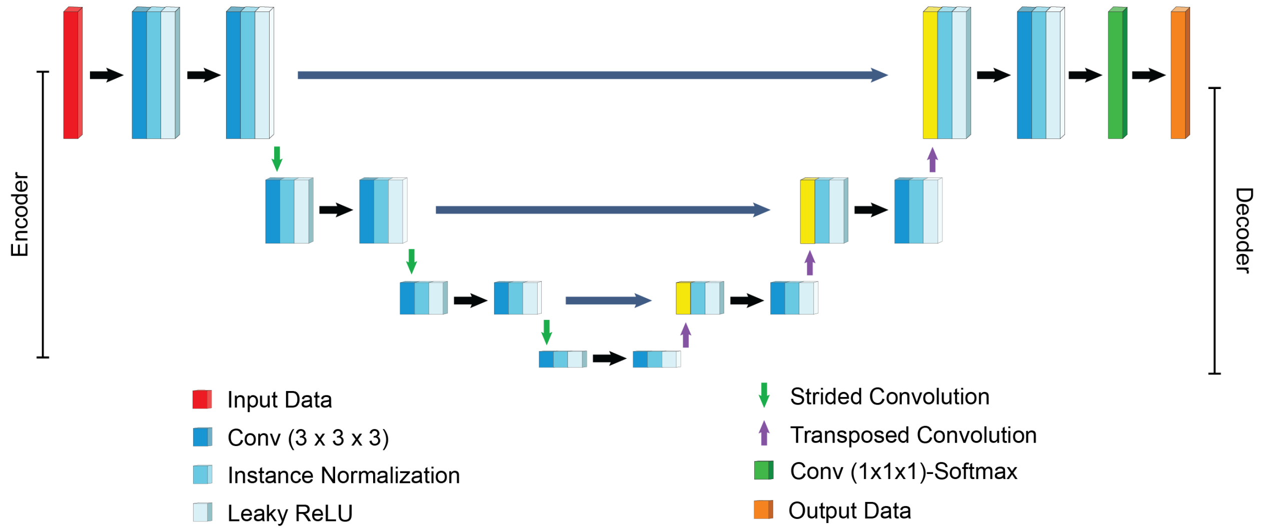

Figure S9: Schematic of the deep learning architecture for NanoMASK. The model is based on a 3D U-NET style architecture utilizing combined inputs of both PET and CT data. The model was trained on 350+ multimodal data volumes for which anatomically correct, 3-dimensional volumes corresponding to key organ systems (heart, lungs, kidneys, liver, spleen, tumor) were manually constructed. It operates on raw PET and CT input data to generate accurate organ contours and automatically produce key pharmacokinetic outputs.

Supplementary Tables

Table 1: Metrics of model accuracy for NanoMASK

	Mean Absolute Relative Error (MARE)							
Organ	%ID/cc	%ID	Region Volume (mm^3)	Mean Intensity (mCi)	Total Intensity (mCi)	SUV_{mean}	SUV_{max}	SUV_{min}
Heart	0.0466 ± 0.1397%	0.0617 ± 0.1411%	0.0542 ± 0.0436%	0.0466 ± 0.1397%	0.0621 ± 0.1409%	0.0466 ± 0.1397%	0.15 ± 0.287%	2.12 ± 6.62%
Lungs	0.108 ± 0.388%	0.14 ± 0.41%	0.0406 ± 0.045%	0.108 ± 0.388%	0.14 ± 0.41%	0.108 ± 0.388%	0.173 ± 0.682%	0.274 ± 0.993%
Kidneys	0.0553 ± 0.0553%	0.123 ± 0.11%	0.0958 ± 0.0771%	0.0553 ± 0.0553%	0.123 ± 0.11%	0.0553 ± 0.0553%	0.283 ± 0.317%	3.92 ± 14.45%
Liver	0.0315 ± 0.0424%	0.051 ± 0.0431%	0.0925 ± 0.2079%	0.0315 ± 0.0424%	0.0779 ± 0.2095%	0.0315 ± 0.0424%	NA	0.572 ± 0.442%
Spleen	0.117 ± 0.124%	0.22 ± 0.5%	0.307 ± 0.543%	0.117 ± 0.124%	0.22 ± 0.499%	0.117 ± 0.124%	0.012 ± 0.0343%	0.773 ± 0.496%
Tumor	0.0812 ± 0.0462%	0.168 ± 0.152%	0.318 ± 0.631%	0.0812 ± 0.0462%	0.286 ± 0.638%	0.0812 ± 0.0462%	0.0322 ± 0.093%	0.925 ± 1.167%
	**Mean Absolute Error (MAE)**							
Heart	0.00424 ± 0.01325	1.77 ± 3.87	0.243 ± 0.596	14.3 ± 52	359000 ± 965000	0.0022 ± 0.0068	0.1550 ± 0.4470	0.00031 ± 0.0007
Lungs	0.0074 ± 0.02883	4.74 ± 14.38	0.306 ± 1.042	24.7 ± 111.4	932000 ± 3520000	0.00388 ± 0.01479	0.2510 ± 0.7990	0.0006 ± 0.0002
Kidneys	0.00377 ± 0.00344	2.27 ± 2.2	0.34 ± 0.643	9.01 ± 9.41	327000 ± 427000	0.00205 ± 0.00196	0.2760 ± 0.2840	0.0002 ± 0.0003
Liver	0.00968 ± 0.01865	15.7 ± 15.8	1.23 ± 3.08	28.3 ± 74.4	2650000 ± 3880000	0.0051 ± 0.00957	0.4661 ± 0.2211	0.0002 ± 0.0002
Spleen	0.0357 ± 0.0431	5.23 ± 6.6	0.372 ± 0.422	89.7 ± 136.9	860000 ± 1427000	0.019 ± 0.023	0.2310 ± 0.7993	0.0142 ± 0.0161
Tumor	0.00248 ± 0.00157	1.08 ± 1.12	1.05 ± 2.32	3.7 ± 3.57	132000 ± 196000	0.00145 ± 0.00101	0.0168 ± 0.0460	0.0012 ± 0.0037
	**Root Mean Squared Relative Error (RMSRE)**							
Heart	0.364 ± 1.091%	0.482 ± 1.102%	0.423 ± 0.341%	0.364 ± 1.091%	0.485 ± 1.1%	0.364 ± 1.091%	1.18 ± 2.24%	16.6 ± 51.7%
Lungs	0.844 ± 3.033%	1.09 ± 3.21%	0.317 ± 0.351%	0.844 ± 3.033%	1.09 ± 3.2%	0.844 ± 3.033%	1.35 ± 5.32%	2.14 ± 7.75%
Kidneys	0.432 ± 0.432%	0.96 ± 0.86%	0.748 ± 0.602%	0.432 ± 0.432%	0.957 ± 0.861%	0.432 ± 0.432%	2.21 ± 2.48%	14.6 ± 12.9%
Liver	0.246 ± 0.331%	0.398 ± 0.337%	0.722 ± 1.624%	0.246 ± 0.331%	0.609 ± 1.636%	0.246 ± 0.331%	3.41 ± 1.22	4.46 ± 3.46%
Spleen	0.912 ± 0.971%	1.72 ± 3.91%	2.4 ± 4.24%	0.912 ± 0.971%	1.72 ± 3.9%	0.912 ± 0.971%	0.0939 ± 0.2676%	6.04 ± 3.87%
Tumor	0.437 ± 0.249%	0.907 ± 0.817%	1.71 ± 3.4%	0.437 ± 0.249%	1.54 ± 3.43%	0.437 ± 0.249%	0.173 ± 0.501%	4.98 ± 6.29%
	**Root Mean Squared Error (RMSE)**							
Heart	0.0331 ± 0.1035	13.8 ± 30.2	1.9 ± 4.65	112 ± 406	2800000 ± 7540000	0.0172 ± 0.0531	0.1210 ± 0.3490	0.0024 ± 0.0062
Lungs	0.0578 ± 0.2252	37 ± 112.3	2.39 ± 8.14	193 ± 870	7280000 ± 27490000	0.0303 ± 0.1156	0.1960 ± 0.2640	0.0518 ± 0.0165
Kidneys	0.0295 ± 0.0268	17.7 ± 17.2	2.65 ± 5.02	70.3 ± 73.5	2550000 ± 3330000	0.016 ± 0.0153	0.2160 ± 0.2220	0.0185 ± 0.0298
Liver	0.0756 ± 0.1457	123 ± 123	9.64 ± 24.09	221 ± 581	20700000 ± 30300000	0.0398 ± 0.0747	0.1683 ± 0.1049	0.01450 ± 0.01750
Spleen	0.279 ± 0.336	40.8 ± 51.6	2.91 ± 3.3	701 ± 1069	6710000 ± 11140000	0.148 ± 0.18	0.1810 ± 0.6240	0.0111 ± 0.0126
Tumor	0.0133 ± 0.0085	5.8 ± 6.03	5.68 ± 12.47	19.9 ± 19.3	708000 ± 1054000	0.00781 ± 0.00544	0.0907 ± 0.0247	0.0066 ± 0.0019
	**Uncertainty at 95% (U95)**							
Heart	2.04e-1	2.51e+2	1.27e+1	1.34e+4	7.86e+12	1.04e-1	1.47e+1	5.79e-2
Lungs	4.45e-1	1.59e+3	2.17e+1	3.90e+4	5.30e+13	2.27e-1	3.86e+1	2.72e-2
Kidneys	5.35e-2	3.48e+2	1.69e+1	5.09e+3	6.51e+12	3.03e-2	4.65e+1	3.44e-2
Liver	2.91e-1	1.53e+4	1.40e+2	5.01e+4	4.29e+14	1.48e-1	1.03e+1	2.09e-2
Spleen	7.37e-1	1.77e+3	1.49e+1	4.93e+5	4.51e+13	3.75e-1	3.28e+1	1.24e-2
Tumor	1.68e-2	4.54e+1	5.67e+1	4.35e+2	5.02e+11	1.07e-2	8.27e+1	4.77e-2

Table 2: Training/Testing splits for NanoMASK and subsetted models

Experiment	Model Name / Type	Training Subset	# Training Data (n)	# Testing Data (n)	Training/Testing Split Within Subset (%/%)	Overall Training/Testing Split (%/%)
Main	NanoMASK Full Model	None	285	71	80/20	80/20
Modality Subset	CT Only	None	285	71	80/20	80/20
Modality Subset	PET Only	None	285	71	80/20	80/20
Timepoint Subset	1h Only	1h p.i.	57	299	80/20	16/84
Timepoint Subset	3.5h Only	3.5h p.i.	57	299	80/20	16/84
Timepoint Subset	6h Only	6h p.i.	57	299	80/20	16/84
Timepoint Subset	24h Only	24h p.i.	57	299	80/20	16/84
Timepoint Subset	48h Only	48h p.i.	57	299	80/20	16/84
Tumor Status Subset	Healthy Only	Healthy Animals	110	246	80/20	31/69
Tumor Status Subset	Tumor-Bearing Only	Tumor-Bearing Animals	175	181	80/20	49/51
Input Organ Subset	(-)Heart	None	285	71	80/20	80/20
Input Organ Subset	(-)Lungs	None	285	71	80/20	80/20
Input Organ Subset	(-)Kidneys	None	285	71	80/20	80/20
Input Organ Subset	(-)Liver	None	285	71	80/20	80/20
Input Organ Subset	(-)Spleen	None	285	71	80/20	80/20
Input Organ Subset	(-)Tumor	None	285	71	80/20	80/20
Modality Subset	1h Only (CT)	1h p.i.	57	299	80/20	16/84
Modality Subset	1h Only (PET)	1h p.i.	57	299	80/20	16/84
Modality Subset	48h Only (CT)	48h p.i.	57	299	80/20	16/84
Modality Subset	48h Only (PET)	48h p.i.	57	299	80/20	16/84
